# Supplementary material for: Chromothripsis during telomere crisis is independent of NHEJ, and consistent with a replicative origin
Source: Genome Res. 2019 May;29(5):737–49. doi: 10.1101/gr.240705.118 (PMC6499312; doi:10.1101/gr.240705.118)
Supplement: Supplemental Material [file supp_gr.240705.118_Supplemental_file_1.zip › contigs/annotated_contigs/DB53/contig.3.DB53_length_567_mean_cov_4.90828924162.docx]

**DB53_length_567_mean_cov_4.90828924162**

```
AGAGC|GGCAGAACAAATTACAGGTAGATTAATTTCCCTTCATCATCAAGCCTTCACACCATCCCTGAACTGTGTCTAAACTTACATGA
      >chr8:133511219-133511411 + E=8e-95
GATAGAAGAATTAATGGACAGTCGATTCAAACCAATGCATTTGGGGCCTGTTTCTTAAAGCAGCCTGTCCTGTACCTTGCTCATATAAA

TGGTGTCTCAAGCAGAGA|TG|GGTGTGGTGGGATATGCCTTCAGCCCCAGCTACTCAGGAGGCTGAGGTAGGAAGATAGCTTGAGCCT
                   >chr8:135054187-135054317 + E=1e-66
GGGAGGTTGAGGCTGCAGTTAGCTGTGATCATGTCACTGTACTATAGGCTGGGTGACAG|AG|TTTTAAATTTTGATGTAGCCCAACTT
                                                            >chr8:133729264-133729508 -
CTCTACTTTTTTTCTTTTTTTGTGGTCTGTGCTTTTGGTGTCACATTCAATAAATCATTGCCAAATCCAATGTCATGAAGTTTCTCACC
 E=4e-135
CATCTTTTGTTGTGTTTTATAGTTTTAGCTCTTACATTTAGGTTTTTAATTCATTTTGAGTTAACCTTTGTATATGGCATAAAGTAATG

CTTCAACTTCATTCTTTTGCGTATAGTGTAGTAAGTTT
```
